# Atomic and Molecular Layer Deposition of Alkali Metal Based Thin Films

**DOI:** 10.1021/acsami.1c17519

**Published:** 2021-11-26

**Authors:** Milad Madadi, Juho Heiska, Jenna Multia, Maarit Karppinen

**Affiliations:** Department of Chemistry and Materials Science, Aalto University, FI-00076 Espoo, Finland

**Keywords:** atomic layer deposition, molecular layer deposition, thin films, alkali metals, metal−organic hybrids, Li-ion battery

## Abstract

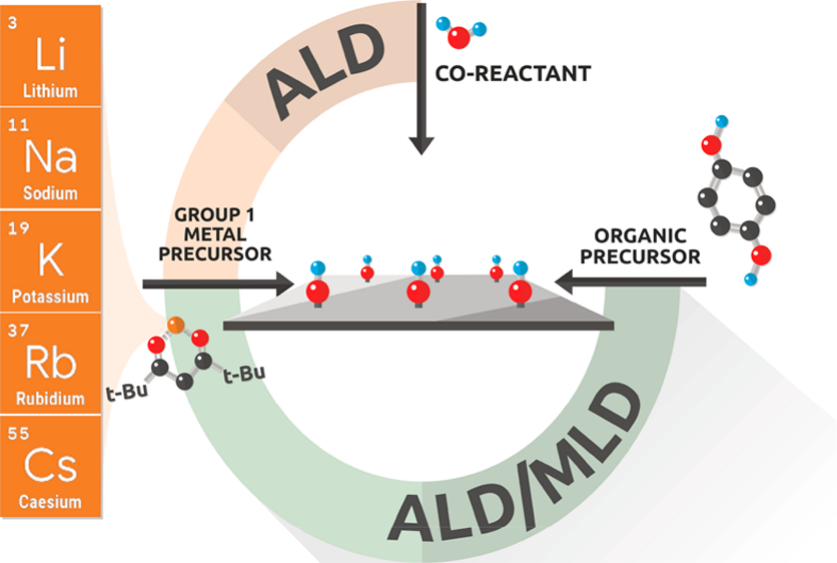

Atomic layer deposition (ALD) is the fastest growing thin-film technology in microelectronics, but it is also recognized as a promising fabrication strategy for various alkali-metal-based thin films in emerging energy technologies, the spearhead application being the Li-ion battery. Since the pioneering work in 2009 for Li-containing thin films, the field has been rapidly growing and also widened from lithium to other alkali metals. Moreover, alkali-metal-based metal–organic thin films have been successfully grown by combining molecular layer deposition (MLD) cycles of the organic molecules with the ALD cycles of the alkali metal precursor. The current literature describes already around 100 ALD and ALD/MLD processes for alkali-metal-bearing materials. Interestingly, some of these materials cannot even be made by any other synthesis route. In this review, our intention is to present the current state of research in the field by (i) summarizing the ALD and ALD/MLD processes so far developed for the different alkali metals, (ii) highlighting the most intriguing thin-film materials obtained thereof, and (iii) addressing both the advantages and limitations of ALD and MLD in the application space of these materials. Finally, (iv) a brief outlook for the future perspectives and challenges of the field is given.

## 1 Introduction

Atomic layer deposition (ALD) is a state-of-the-art gas-phase fabrication technique for high-quality inorganic thin films, and owing to its many superior features, it has been the fastest growing thin-film technology in microelectronics already for decades.^1−7^ The first developments of the ALD technique date back to the 1960s and 1970s,^1,2^ and the semiconductor industry adopted the technique for high-*k* dielectrics in the 2000s, but in recent years, it has been emerging in other application areas too, such as solar power, light-emitting diodes (LEDs), and optics. While the periodic table of elements for which ALD processes have been developed already covers most of the elements,^8^ the processes used in industry so far involve only a limited number of them (viz., Al, Ti, Zn, Hf, O, S). Another fact related to the elements involved in ALD is that the alkali metal group has remained very little explored until recently.

The field of alkali metal ALD was pioneered by Putkonen et al.^9^ in 2009. This first paper focused on the potential ALD precursors for lithium, and it instantly underlined the challenges related to alkali metal ALD in general.^10^ Alkali metals are monovalent and their precursors thus mostly monoleptic. This means that the simple surface chemistry principles commonly sketched for protype ALD processes, based on multileptic precursors capable of forming multiple covalent bonds, are not directly met in the case of alkali metals. For example, we may need to extend the self-saturating surface chemisorption concept central in conventional ALD to self-saturating surface physisorption.^11−13^ Moreover, the most widely investigated alkali metal, lithium, is one of the lightest elements, which makes it extremely mobile within the growing film.

The chemical bonds forming in alkali metal ALD bear unique features as well. Namely, the bonds formed by alkali metals through their spherical outer s orbitals are characteristically nondirectional ionic bonds. Such bonds are free from electron pair repulsion rules and preferred bond directions, which has been considered a benefit when the goal is to grow in situ crystalline films.^14^ On the other hand, alkali metals are basic and hygroscopic and prone to rapidly reacting into hydrates and carbonates; this often makes the ex situ characterization of, for example, alkali metal oxide and hydroxide films challenging. An archetypal example is the ALD-grown hygroscopic LiOH films showing a so-called “water-reservoir effect” and ultrahigh and nonreproducible growth rates.^15,16^

In recent years, a branch of the ALD technology known as molecular layer deposition (MLD), based on organic precursors instead of metal-bearing precursors,^17^ has been strongly emerging. Moreover, mixing the ALD and MLD cycles is possible such that, in a mixed ALD/MLD process, a metal-bearing precursor is combined with an organic precursor to deposit metal–organic thin films.^18−22^ The first alkali-metal-based ALD/MLD-grown metal–organic thin films were reported in 2016.^23^ An exciting feature of these alkali-metal-based hybrid thin films was that many of them were in situ crystalline. Another attractive result was noticed soon after: Through ALD/MLD, it is possible to stabilize even compositions/structures not known before.^24^ The first example was the Li-quinone films grown from lithium hexamethyl disilazide and 1,4-dihydroxybenzene (hydroquinone) precursors.^25^ According to DFT calculations, the new crystal structure contains the Li^+^ cations in a coordinatively unsaturated three-fold coordination, which explains why this compound had not been reported before, as such undercoordinated metal sites tend to accommodate solvent molecules and are thus difficult to obtain through any solution-based synthesis route.

**Figure 1:**
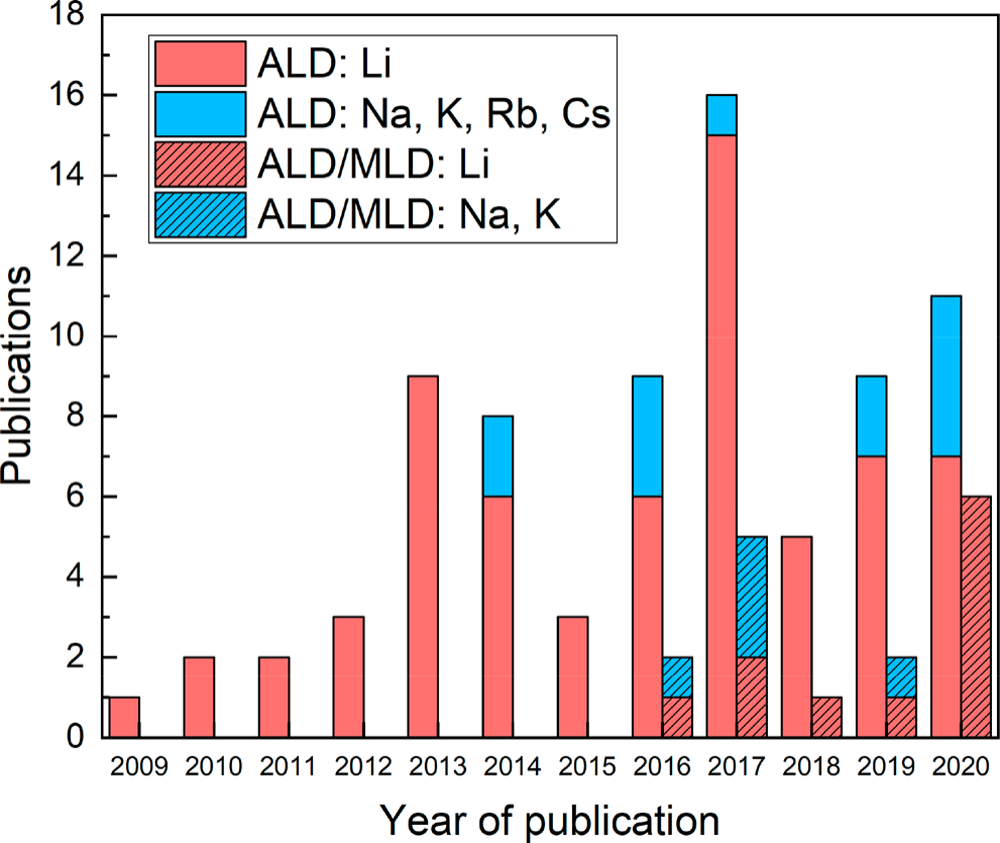
Annually published ALD and ALD/MLD articles involving alkali metals, as of the end of 2020.

In Figure 1, we plot the number of annually published alkali-metal-based ALD and ALD/MLD papers. It is clear that lithium is the leader among the alkali metals. The motivation is naturally in the Li-ion battery (LIB) technology, where ALD and MLD are seen as the most promising thin-film techniques for high-quality electrode and electrolyte materials and coatings.^13,26−29^ Along with this, there is also a growing interest in sodium- and potassium-based thin films in battery applications and other sustainable energy technologies, such as solar cells, thermoelectrics, and piezo- and ferroelectric devices.

Despite the rapidly growing interest in the topic, there are no comprehensive reviews on the use of ALD and MLD techniques for alkali-metal-based thin films. In an early review by Nilsen et al.,^13^ the challenges in the ALD for Li-ion microbatteries were discussed with an emphasis on the Li-based ALD processes. More recently, Li-ALD solid-electrolyte materials were covered by Meng,^29^ while Sønsteby et al.^30^ focus on alkali metal (Li, Na, K, Rb, and Cs) *tert*-butoxides as precursors in ALD in their recent review.

In this review, we present a comprehensive account of the current state of research in the field, comprising not only the entire literature on alkali-metal-based ALD processes but also the new alkali-containing metal–organic materials realized through ALD/MLD. Our intention is to highlight the wide range of processes and materials that can be fabricated by ALD and ALD/MLD, and to illustrate both the advantages and limitations of ALD in the application space of these materials. We start with a brief description of the basics of the ALD and ALD/MLD techniques (Section 2), and a summary of the alkali metal precursors used (Section 3). Then, particular efforts are made to summarize and discuss the ALD (Sections 4 and 5) and ALD/MLD (Section 6) processes so far developed for alkali-metal-based inorganic and metal–organic thin films, respectively. Finally, after addressing these main issues, we devote a short section to the application potential of these thin-film materials (Section 7) and a brief outlook for the future perspectives and challenges in this scientifically exciting and industrially promising field (Section 8).

## 2 ALD and MLD Techniques in Brief

Atomic layer deposition is a chemical gas-phase thin-film fabrication technique, where the precursors are introduced as gas pulses into the reactor, one at a time. This alternating pulsing of the different precursors makes the ALD approach different from the parent chemical vapor deposition (CVD) technique, in which the precursors are simultaneously supplied. The ALD technique has its origins both in the USSR since the 1960s^31^ and in Finland since the 1970s,^2^ the latter efforts leading to the first industrial applications in electroluminescence displays at an amazingly fast pace.^2−5^

In a typical binary ALD process, two different gaseous/vaporized precursors are sequentially pulsed and purged out of the reaction chamber with prespecified time intervals. The precursor pulse and purge times are selected so that the precursors have enough time to chemisorb onto the substrate surface and react with the available surface groups for full surface coverage. Another important feature is the self-limitation of the surface reactions, such that the chemisorption of one precursor is limited to a monolayer: The reaction only continues after the excess precursor molecules from the gas phase are purged out and the second precursor is delivered to the reaction chamber. As the two precursors are not present at the same time in the chamber, no unwanted gas-phase reactions occur. This unique growth process leads to highly uniform and conformal thin films with atomic-level control for both the film thickness and composition.^32,33^ The high degree of conformality has been assessed by depositions on test structures with aspect ratios as high as 10,000:1.^34^

**Figure 2:**
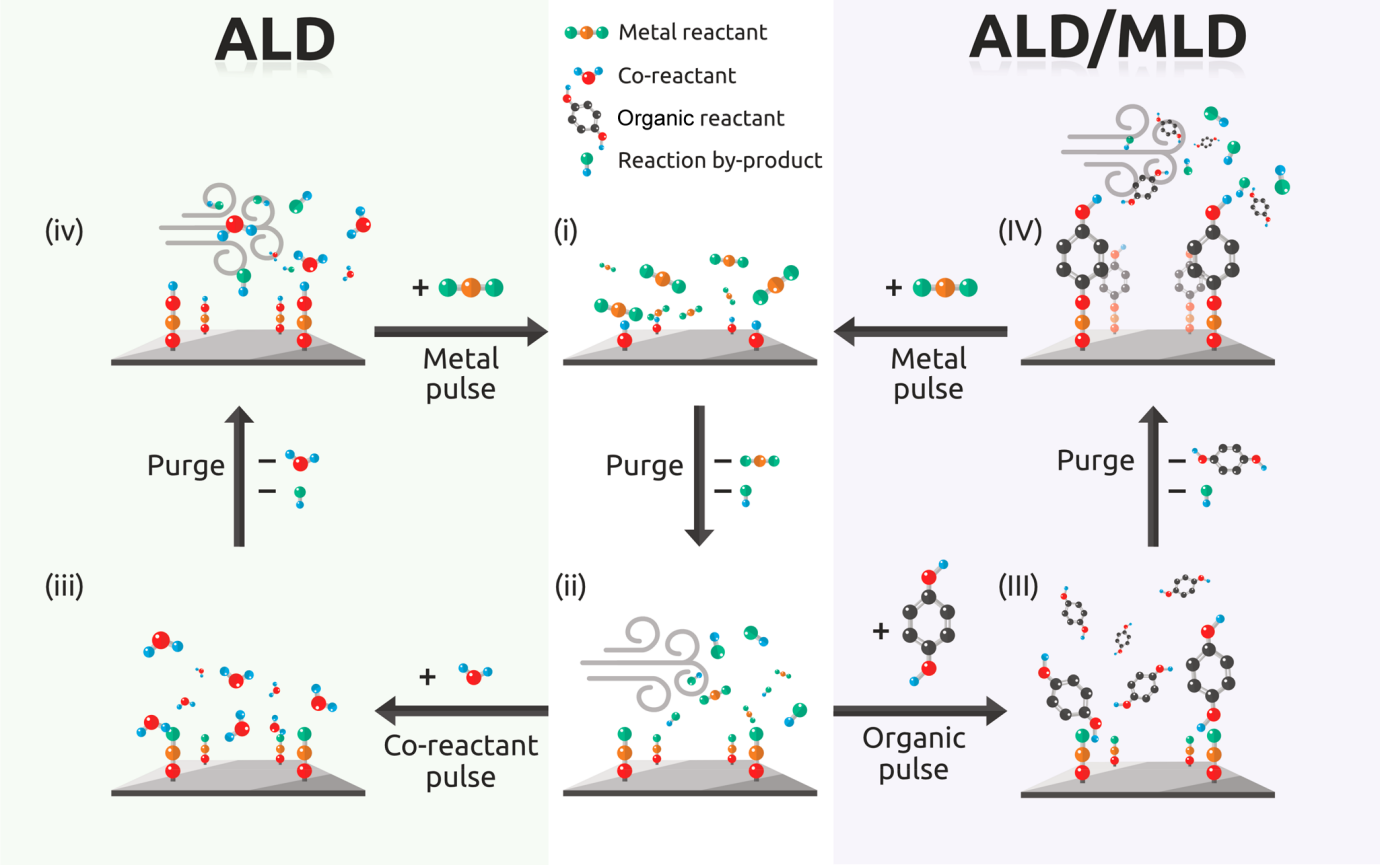
ALD (on the left) and ALD/MLD (on the right) precursor pulsing cycles for the deposition of inorganics (e.g., metal oxide) or metal–organics, respectively: (i) metal precursor, (ii) purge, (iii) coreactant, such as water in ALD or hydroquinone in ALD/MLD, and (iv) purge. Note that the first two steps, (i) metal precursor and (ii) purge, are common for both processes.

The two (or more) precursor sources (gas, liquid, or solid) and the substrate are placed in or connected to the reactor. If necessary, the different sections of the reactor are heated to specific temperatures to assist with the precursor delivery and to realize the required reaction temperature on the substrate surface. Also, the reactors typically operate under reduced pressure (mbar range), and inert gas (N_2_ or Ar) is used as both a precursor carrier gas and a purge gas to free the reactor chamber from any unreacted precursor molecules and the reaction byproducts after each precursor pulse. The sequence of the two precursor pulses and the consecutive inert gas purges form a so-called ALD cycle: (i) precursor 1 (metal precursor), (ii) purge, (iii) precursor 2 (coreactant), and (iv) purge; see Figure 2. This cycle is repeated until the desired film thickness is reached.

Prototypical ALD processes include those for binary metal oxides (e.g., Al_2_O_3_, HfO_2_, TiO_2_, ZnO)^35,36^ and sulfides (e.g., ZnS),^2^ but ternary and even quaternary processes are possible as well, though more challenging to optimize.^37−39^ Organometallic compounds such as trimethylaluminum (TMA) and diethylzinc (DEZ) or metal halides such as TiCl_4_ or HfCl_4_^40^ are usually used as the metal precursors, as they have much lower sublimation/evaporation temperatures than, for example, elemental metals, thus allowing for the reactor to be operated at a reasonably low temperature. The second precursor is then typically the source of oxygen (e.g., H_2_O, O_3_), sulfur (e.g., H_2_S, S),^2,41^ or nitrogen (e.g., NH_3_).^42^

While the standard ALD technique involves only inorganic thin-film materials, its counterpart for organic thin films was introduced in 1991;^17^ this technique based on two different organic precursors was termed MLD. Similarly to the parent ALD, in MLD the organic precursors are sequentially pulsed into the reactor for the growth of organic polymeric films (e.g., polyimides and polyamides) with high precision.^43^ Moreover, since both ALD and MLD are modular in principle, it is straightforward to combine them for the growth of hybrid metal–organic thin films.^18−22^ The combined ALD/MLD technique for the metal–organics involves a metal precursor similar to those used in ALD and one organic precursor that matches with the metal precursor regarding the chemical and thermal properties. To facilitate the gas–surface reactions, the two precursors need to be mutually reactive. This requires that the metal precursor has reactive ligands attached to the metal ion and that the organic precursor has reactive functional groups attached to the organic backbone. An ALD/MLD cycle thus consists of the following pulses: (i) metal precursor, (ii) purge, (iii) organic precursor, and (iv) purge, see Figure 2.

The history of the ALD/MLD technique for metal–organic thin films is barely longer than a decade;^18,19^ nevertheless, already tens of ALD/MLD processes have been developed. Initially, the most conventional metal (Al, Ti and Zn) and organic (e.g., ethylene glycol) components were investigated, but in recent years, a rich variety of hybrid materials with different metal (alkali metal, alkaline earth metal, 3d transition metal, lanthanide) and organic (allyl, aryl, pyridine, nucleobase, etc.) constituents have been explored.

Like in ALD, the metal–organic thin films can be accurately thickness and composition controlled. Another feature common to both ALD and ALD/MLD is that the as-deposited films are usually amorphous, not crystalline. However, in 2016, the first in situ crystalline copper-terephthalate films of the well-known metal–organic framework (MOF)-2 structure were realized.^44^ These were soon followed by many other in situ crystalline films, examples including several films based on alkali metals as well.^14,23,25^

**Figure 3:**
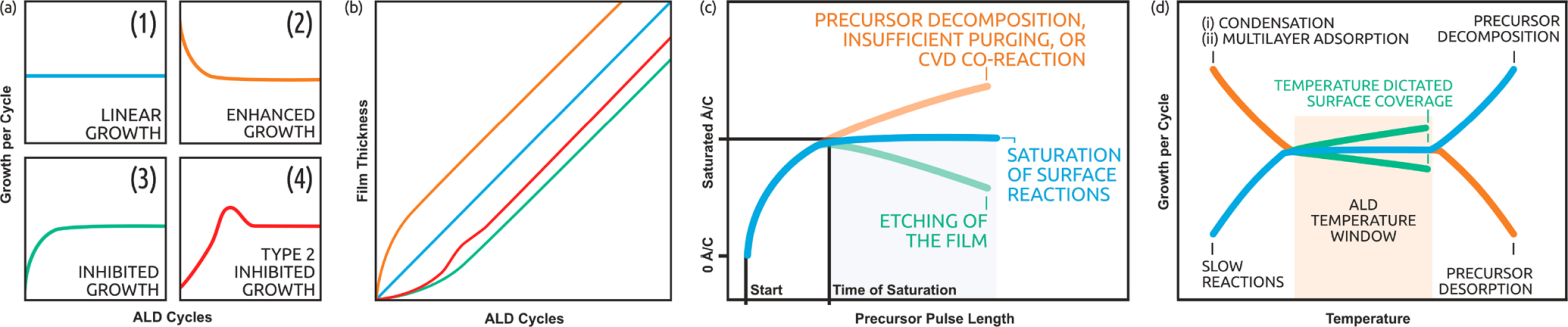
Typical ALD and ALD/MLD process parameter investigations: Dependence of film growth on (a, b) the number of deposition cycles, (c) precursor pulse length, and (d) deposition temperature.

In both ALD and ALD/MLD, the growth process is typically monitored and evaluated by following the film growth rate as a function of various deposition parameters (precursor and purge pulse lengths, deposition temperature, etc.), see Figure 3. The common expression for the growth rate is the so-called growth-per-cycle (GPC) value, calculated from the total film thickness divided by the number of precursor pulsing cycles applied, and given in the units of Å per cycle (Å/c). For ideal ALD and ALD/MLD processes, a saturation behavior is expected wherein the GPC value increases together with pulse lengths but remains constant independent of the number of deposition cycles; this latter requirement is often expressed as a linear dependence of film thickness on the number of deposition cycles.

## 3 Alkali Metal Precursors for ALD

Precursor chemistry is one of the cornerstones in both ALD and MLD.^4,45^ The main challenge in the precursor design is to find the optimal balance between their reactivity and thermal stability. Volatility is a crucial requirement as well, and in principle, gaseous and liquid precursors are favored for the efficient and stable precursor supply. However, numerous well-performing solid precursors have been successfully developed and applied as well. The precursor development is centered around finding the optimal ligands, in terms of size and chemistry. For the metal precursor, both homoleptic and heteroleptic systems have been utilized. In general, the ligand variety includes, for example, different halides, alkyls, alkoxides, alkyl amides, cyclopentadienyls, and metallocenes.

**Figure 4:**
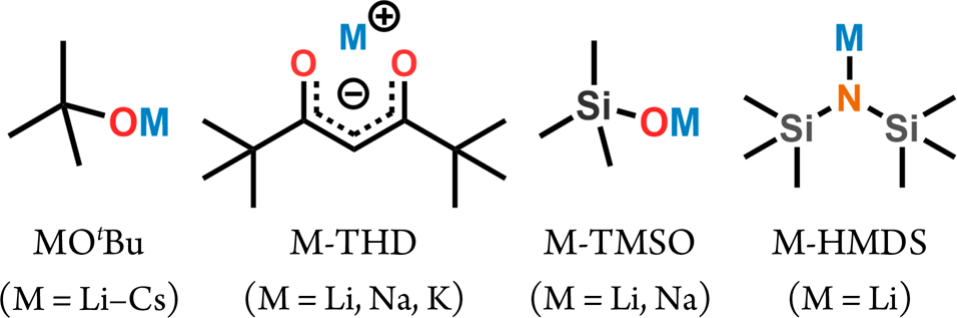
Alkali-metal precursors used for ALD and ALD/MLD.

For alkali metals, all the precursors so far exploited are solid materials, and the most common precursor type has been the *tert*-butoxide MO^*t*^Bu (M = alkali metal), in particular for the alkali metals other than lithium.^30^ For Li, the ALD literature is more extensive and also the precursor variety wider.^13^ Other common alkali metal precursors include M-THD (THD = 2,2,6,6-tetramethyl-3,5-heptanedionate), Li-HMDS (HMDS = hexamethyldisilazide or bis(trimethylsilyl)amide), and M-TMSO (TMSO = trimethylsilanolate). Molecular structures of these precursors are displayed in Figure 4. A common feature among the precursors is the presence of relatively bulky *t*-Bu or TMS (trimethylsilyl) groups, whose steric hindrance lowers the interactions between the molecules. This increases their volatility and suitability for the ideal ALD film growth, where reactions other than those occurring on the surface-to-be-coated should be avoided. Another common and unavoidable feature (due to the reactivity of the metal component itself) among the alkali metal precursors is their instability in air, but the extent of it varies by precursor.

The utilization of alkali metal *tert*-butoxides in ALD has been reviewed in detail by Sønsteby et al.^30^ The MO^*t*^Bu molecules form oligomer clusters in both solid and gas phases, primarily hexamers in case of Li and Na and tetramers with the heavier alkali metals. This clustering contributes to the vapor pressures of the precursors. The sublimation temperature used for their purification increases for heavier alkali metals (M): 110 °C for Li, 140 °C for Na, 150 °C for K, 190 °C for Rb, and 200 °C for Cs. The MO^*t*^Bu compounds are increasingly hygroscopic in particular for the heavier alkali metals, and they react with water in ambient air to form alkali metal hydroxides and *tert*-butanol. This hydroxide tends to appear as a transparent encapsulating layer around the precursor particles, which may be an issue due to the low volatilities of alkali metal hydroxides at typical deposition temperatures. The *tert*-butanol is present in the precursor in the form [MO^*t*^Bu]_*x*_·[Bu^*t*^OH], although thermogravimetric (TG) analysis has shown that it is released upon heating to around 50–100 °C.^46^ The *tert*-butanol can ignite if the exothermic reaction proceeds quickly, so overt exposure to water should be avoided. Despite their hygroscopicity, the MO^*t*^Bu precursors have worked in an acceptably reproducible manner when handled in inert conditions up until loading them into the ALD reactor and only being exposed to air during the loading process.^30^

For lithium-based films, Li-THD has been used as a precursor since the first alkali-metal-related ALD experiments in 2009.^9^ Additionally, in ALD/MLD, different M-THD precursors have been successfully used in combination with organic precursors.^22^ Due to its lower reactivity compared to, for example, LiO^*t*^Bu, Li-THD is relatively stable in air but requires a higher vaporization temperature (typically 175–200 °C). Additionally, a reactive second precursor is needed, such as ozone in ALD^13^ or a carboxylic acid in ALD/MLD.^23^ The THD ligand is bulky, which is likely to result in relatively low growth rates when using these precursors.^47^

The TMSO ligand is otherwise similar to O^*t*^Bu, but the central atom is silicon in the former and carbon in the latter. It should be noted that the Si–C bond is longer (and more polar) than the C–C bond, which may affect the ligands’ behavior. In early ALD experiments with Li-TMSO, the precursor was heated to 165 °C for sublimation and combined with H_2_O and CO_2_ to obtain Li_2_CO_3_ films, as well as with O_3_ and H_2_O (in that order) to obtain Li–Si–O films.^48^ On the other hand, in the case of the sodium counterpart, Na-TMSO, significant film-thickness gradients were observed.^49^

**Figure 5:**
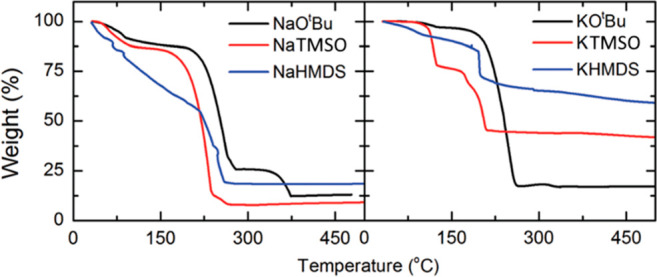
TG curves for various alkali-metal precursors.^49^

The HMDS-based precursors are different from the other alkali metal precursors in that, in these molecules, the metal is bound to nitrogen instead of oxygen. However, so far, positive results have been reported only for Li-based films. This may be related to the precursors’ different oligomeric structures and chemical characteristics:^49−51^ In solid/gas phases, Li-HMDS is trimeric/dimeric, Na-HMDS is polymeric/monomeric, and K-HMDS is dimeric/unknown, respectively. Moreover, K-HMDS is ionic, while the other two are covalent. The effects of these differences between HMDS and other precursors for Na and K have been investigated using TG (Figure 5). For Li-based films, the use of the Li-HMDS precursor has provided a way to supply silicon into the film, though the latter worked only with O_3_, and not with H_2_O^13^ or organic precursors. Li-HMDS decomposes in water but tolerates short to moderate handling times in air, and sublimes at comparatively low temperatures (60–75 °C), which has made it relatively popular since its introduction as an ALD precursor.^13^

Overall, when the need is for a highly stable and easily handled precursor, the M-THD compounds would be the most apparent choice, if the necessitated higher deposition temperature (>200 °C) is not an issue. If higher reactivities are looked for, the most thoroughly investigated MO^*t*^Bu compounds could be a great option, even though they require extra care in handling. For processes at extremely low temperatures (even as low as 80 °C), Li-HMDS has already proven its potential, but the other M-HMDS compounds need yet to be explored. Finally, if the presence of silicon is not considered harmful, the M-TMSO precursors could also prove useful.

## 4 Lithium-Based ALD Processes

In the very first lithium-based ALD experiments in 2009,^9^ the following Li precursors were tested: Li-THD, LiO^*t*^Bu, LiCp (Cp = cyclopentadienyl), *n*-BuLi (*n*-butyllithium), and lithium dicyclohexylamide. The most promising results were obtained for Li-THD, which together with O_3_ produced Li_2_CO_3_, and for LiO^*t*^Bu, which with H_2_O yielded thin films with lower carbon content. When applied with water, Li-THD resulted in no films, while LiCp and *n*-BuLi had issues with reproducibility and ALD-type growth, respectively.^9^ In later works, the majority of Li-containing films have been deposited using LiO^*t*^Bu, while Li-THD and Li-HMDS are the second and third most commonly utilized Li precursors. The processes are summarized in Table 1 and discussed here by categorizing them by the number of precursors employed in the process. An exception is the Li_3_PO_4_ and LiP_*x*_O_*y*_N_*z*_ (LiPON) films for which multiple elements (P, O, N) have been incorporated into the film even from a single precursor;^52−54^ these processes are discussed in a designated section at the end of this section.

**Table 1:** Li-Containing Thin Films Deposited with ALD^a^

material	precursor 1	precursor 2	precursor 3	precursor 4	GPC (Å/c)	*T* (°C)	ref
Li_2_O	LiO^*t*^Bu	H_2_O			0.1	225–300	(9, 58)
		^P^O_2_			0.8	50–300	(57)
	Li-HMDS	H_2_O/^P^O_2_/O_3_			0.5–5.6	200–350	(59)
Li_2_O/LiOH	LiCp/*n*-BuLi	H_2_O			–	225	(9)
LiOH	LiO^*t*^Bu	H_2_O			0.1–1.5	50–250	(15, 57, 58, 60, 61)
Li_3_N	Li-HMDS	NH_3_			1.0	167–332	(65)
Li_2_S	LiO^*t*^Bu	H_2_S			1.1	150–300	(66)
LiF	Li-THD	TiF_4_			1–1.4	250–350	(67, 68)
	LiO^*t*^Bu	TiF_4_			1–1.7	200–300	(69−71)
		HF			0.8–0.9	150	(72)
		NH_4_F			0.5	225	(73)
	Li-HMDS	HF			0.2	100–150	(74−77)
		^P^SF_6_			0.4	150	(78)
(LiF–CF_*x*_)	LiO^*t*^Bu	Hfac			1.7	220	(71)
Li_2_CO_3_	Li-THD	O_3_			0.1–0.3	185–225	(9, 55, 56)
	LiO^*t*^Bu	^P^O_2_			0.4–0.8	50–250	(57, 58)
		H_2_O	CO_2_		0.2–0.8	100–300	(57, 62−64)
	Li-HMDS	H_2_O	CO_2_		0.4	186	(65)
	Li-TMSO	H_2_O	CO_2_		0.3–0.5	200–300	(48)
LiP_*x*_O_*y*_	LiO^*t*^Bu	Me_3_PO_3_	H_2_O^a^		2	175	(79)
		PO(OMe)_3_			0.5–1.0, 6.7^80^	225–350	(80−84)
			H_2_O		0.2	275	(64, 85−87)
	Li-HMDS	PO(OMe)_3_			0.4–1.3	275–350	(81)
LiAl_*x*_O_*y*_	LiO^*t*^Bu	AlMe_3_	O_3_		–	150	(88)
		H_2_O	AlMe_3_	O_3_	1.4–2.8	225	(89, 90)
			AlMe_3_	H_2_O	1.5–2	225	(15, 91, 92)
	Li-TMSO	H_2_O	AlMe_3_	O_3_	2.0	175–300	(48, 93)
LiAl_*x*_F_*y*_	LiO^*t*^Bu	AlCl_3_	TiF_4_		1.1	250	(69)
LiAl_*x*_S_*y*_	LiO^*t*^Bu	Al(NMe_2_)_3_	H_2_S		0.5	150	(94)
LiCo_*x*_O_*y*_	LiO^*t*^Bu	CoCp_2_	^P^O_2_		0.6	325	(95, 96)
LiLa_*x*_O_*y*_	Li-THD	La-(THD)_3_	O_3_^a^		0.2–0.3	225	(9)
LiMn_*x*_O_*y*_	Li-THD	Mn-(THD)_3_	O_3_^a^		0.2	225	(97)
	LiO^*t*^Bu	Mn-(THD)_3_	O_2_^a^		2.5	400	(98)
	LiO^*t*^Bu	H_2_O	Mn-(THD)_3_	O_3_	low^97^	250	(97, 99)
	Li-HMDS		Mn(EtCp)_2_	H_2_O		200 (HMDS)	
LiNb_*x*_O_*y*_	LiO^*t*^Bu	Nb(OEt)_5_	H_2_O^a^		1.8–2.9	235	(100)
	Li-HMDS	Nb(OEt)_5_	H_2_O^a^		0.4–0.6	235	(16)
LiNi_*x*_O_*y*_	Li-HMDS	NiCp_2_	^P^O_2_		1.5	300	(101)
LiSi_*x*_O_*y*_	Li-HMDS	O_3_			0.3–1.7	150–400	(11, 12)
	LiO^*t*^Bu	Si(OEt)_4_	H_2_O^a^		0.8–1.4	225–300	(102)
	Li-TMSO	O_3_			1.6	225	(48)
LiSn_*x*_O_*y*_	Li-HMDS	H_2_O/^P^O_2_/O_3_	SnEt_4_	^P^O_2_/O_3_	0.6–3.3	250 (H_2_O); 300	(59, 101)
LiTa_*x*_O_*y*_	LiO^*t*^Bu	Ta(OEt)_5_	H_2_O^a^		0.8;^103^ 2.1^104^	225	(103−106)
LiTi_*x*_O_*y*_	LiO^*t*^Bu	TiCl_4_	H_2_O^a^		2.0	225	(107)
		Ti(O^*i*^Pr)_4_			0.3		
	LiO^*t*^Bu	Ti(NMe_2_)_4_	H_2_O		0.3	300	(108)
LiAl_*x*_Si_*y*_O_*z*_	LiO^*t*^Bu	AlMe_3_	Si(OEt)_4_	H_2_O^a^	1.2^61^	290	(61, 109)
		SiO_2_	AlMe_3_	H_2_O^a^	–	290	(110)
LiB_*x*_C_*y*_O_*z*_	LiO^*t*^Bu	(Me_2_CHO)_3_B	O_3_		0.6	200–260	(111)
LiFe_*x*_P_*y*_O_*z*_	LiO^*t*^Bu	FeCp_2_	P(OMe)_3_O	H_2_O^a^	–	300	(112)
		FeCl_2_	P(OMe)_3_O	H_2_O^a^	2	400	(98)
LiLa_*x*_Ti_*y*_O_*z*_	LiO^*t*^Bu	La-(THD)_3_ + O_3_	TiCl_4_	H_2_O^a^	0.4–0.5	225	(9, 113)
LiLa_*x*_Zr_*y*_O_*z*_	LiO^*t*^Bu	La(*^i^*PrFMD)_3_	Zr(NMe_2_)_4_	O_3_^a^	–	225	(114)
LiNi_*x*_Si_*y*_O_*z*_	Li-HMDS	NiCp_2_	^P^O_2_		1.4	300	(115)
LiP_*x*_O_*y*_N_*z*_	LiO^*t*^Bu	PO(NH_2_)(OEt)_2_			0.2–0.9	200–300	(52, 53)
	Li-HMDS	PO(NH_2_)(OEt)_2_			0.6–0.7	270–310	(52, 54)
	LiO^*t*^Bu	H_2_O	PMe_3_	^P^N_2_	0.8	200–275	(85−87,116)
		NH_3_	P(NMe_2_)_3_	O_2_	0.7–2.1	350–500	(117)
LiTi_*x*_P_*y*_O_*z*_	LiO^*t*^Bu	PO(OMe)_3_	Ti(O^*i*^Pr)_4_	H_2_O	0.9	250	(118)

a Denotes an oxygen precursor pulsed after every non-O precursor.

### Binary Li-Based Processes

A wide variety of Li-based materials have been deposited using just two precursors. Particularly in the earlier years of Li ALD, the experiments mostly focused on testing different lithium precursors in combination with the most common oxygen precursors, H_2_O and O_3_. These depositions typically yielded LiOH, Li_2_O, or Li_2_CO_3_.^13^ Picking the right combination of precursors made a significant difference in the films’ growth, elemental composition, and structure. Notably in many cases, O_3_ was found to react with C-containing^9,55−58^ and Si-containing^11,12,48^ precursors in a way that incorporated those elements into the film. In later experiments, the same was seen using O_2_-plasma with C-containing precursors.^57−59^

Several groups have combined LiO^*t*^Bu and H_2_O to deposit films identified as LiOH^57,58,60,61^ or Li_2_O,^57,58^ with higher deposition temperatures (≥240 °C) favoring the latter.^58^ Most of the depositions were carried out at 200–250 °C, resulting in a wide range of growth rates within 0.1–1.5 Å/c. In most cases, these have been rather preliminary tests before the addition of more precursors to the process. The composition of the as-deposited films often remained ambiguous due to the tendency of Li_2_O to react with H_2_O and CO_2_ in ambient air to form LiOH and Li_2_CO_3_.^9^ Cavanagh et al.^62^ used LiO^*t*^Bu and H_2_O to deposit LiOH and reported a mass growth rate of 12.7 ng/cm^2^ per cycle using a QCM (quartz crystal microbalance); this mass growth rate translates into ∼0.9 Å/c, assuming a LiOH density of 1.46 g/cm^3^ for the films. They also proposed a simple reaction mechanism wherein the LiO^*t*^Bu precursor bonds with a LiOH surface and reacts then with the subsequently pulsed H_2_O to form LiOH, and HO^*t*^Bu as the (departing) side product.^62^ Another group concurred with this mechanism and saw nonlinear growth during the first 30 cycles of LiOH deposition, which they ascribed to initial nucleation and to the hygroscopicity of LiOH.^15^ A later study discussed the difficulties in determining the true growth rates for this system because of the immediate reaction of LiOH in air to form Li_2_CO_3_.^61^

For the deposition of Li_2_CO_3_ films, multiple precursor combinations and processes have been reported. These include Li-THD and O_3_,^9,55,56^ LiO^*t*^Bu and O_2_-plasma (^P^O_2_),^57,58^ as well as one of the several lithium precursors paired with H_2_O and CO_2_.^48,57,62−65^ In binary processes, ^P^O_3_ and ^P^O_2_ were found to decompose the organic moieties of the lithium precursor, resulting in carbon incorporation into the film. This was not always a complete process; for example, at higher temperatures, the use of ^P^O_2_ resulted in significant Li_2_O and LiOH formation.^57^ Most of the Li_2_CO_3_ depositions have been carried out at 150–250 °C, yielding crystalline films at 0.1–0.8 Å/c; however, the roughness of the crystalline films made the thickness measurements challenging.^56,65^

Lithium silicate films have been deposited by binary processes with O_3_ in similar manner to some Li_2_CO_3_ films, utilizing the capacity of O_3_ to decompose the lithium precursor. In this case, the precursor was the Si-containing Li-HMDS.^11,12^ The stoichiometry of the resultant LiSi_*x*_O_*y*_ film was found to depend on the deposition temperature such that, at higher temperatures, more HMDS ligand fragments remained adsorbed on the surface of the film. Along with these HMDS fragments, Si was then incorporated into the films, resulting in the higher Si/Li ratios. At 250 °C, the stoichiometry was close to Li_2_SiO_3_, and films grew at 0.8 Å/c.^11,12^

Lithium fluoride is of interest for ultraviolet optics due to its large optical band gap.^67,74−77^ It is also one of the components of the spontaneously forming solid-electrolyte interphase (SEI) layers in LIBs,^119^ which is why it has been deposited for the protection of battery electrodes.^69,71,72^ The reported studies on LiF ALD processes^67,68,73,76,77^ cover a wide range of deposition temperatures (100–350 °C) and a variety of Li precursors, with the fluorine source generally being TiF_4_ or HF, and they resulted in crystalline LiF films. Initially, to achieve more uniform film growth, LiF films were produced using MgF_2_ (from Mg-THD and TiF_4_) as an intermediate step, and then Mg-THD was replaced with Li-THD in a subsequent metal precursor pulse.^67^ Deposition of LiF films directly was eventually accomplished with careful temperature control and large Li-THD doses.^68^ The microroughness and thus the refractive index of the films have been found to depend on the deposition temperature.^77^ Recently, a third fluorine precursor, hexafluoroacetylacetone (HFAC), was employed to deposit porous, hybrid LiF-CF_*x*_ films with improved Li^+^-ion conductivity compared to the purer LiF.^71^

In addition to LiF, other nonoxide Li compounds have also been grown with ALD. For example, Li_2_S is interesting as it could serve as an alternative to the Li-metal anode in batteries. The Li_2_S films deposited from LiO^*t*^Bu and H_2_S at temperatures as low as 150 °C were found uniform and conformal.^66^ Based on QCM results, the following reaction mechanism of exchanging places for protons and Li-ions was proposed.
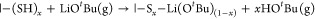

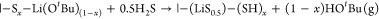


Also, uniform and linearly growing amorphous Li_3_N films were successfully deposited at 167 °C from Li-HMDS and NH_3_ precursors, using MoN_*x*_ as both an adhesion layer on the substrate and a capping layer for protecting the films from reactions in ambient air. Without the adhesion layer, the depositions were found unsuccessful on several substrate types. A deposition temperature of 332 °C further yielded crystalline Li_3_N films.^65^

### Ternary Li-Based Processes

There are many Li ALD processes based on more than two different precursors; in most of these processes, one of the precursors is an oxygen source (H_2_O, O_3_, or both), and the most common product is a mixed oxide. As mentioned earlier, Li_2_CO_3_ films have been deposited with a ternary process by pulsing H_2_O and CO_2_ after the lithium precursor.^48,57,62−65^ This mimics the natural process of LiOH conversion into Li_2_CO_3_ upon exposure to ambient CO_2_. However, performing the exposure layer-by-layer in an ALD reactor allows for a better-controlled, more thorough conversion and enables the deposition of Li_2_CO_3_ without the use of strong oxidizers such as O_3_ or O_2_-plasma. Most of these depositions have been carried out at 150–250 °C, the degree of crystallinity typically increasing at higher deposition temperatures.^57,65^ As the Li precursor, LiO^*t*^Bu,^57,62−65^ LiHMDS,^65^ and LiTMSO^48^ have been used, yielding growth rates in the range of 0.2–0.8 Å/c.

For lithium silicates, two ternary processes have been reported.^48,102^ One of them is like the previously discussed Li-HMDS + O_3_ process (where Li-HMDS provides both Li and Si), but supplemented with H_2_O as the third precursor to provide −OH groups for enhancing the reaction of Li-TMSO with the surface.^48^ In the other process, Li and Si are supplied separately via LiO^*t*^Bu and Si(OEt)_4_ (tetraethylorthosilane, TEOS), and H_2_O is pulsed after each of these. In other words, LiSi_*x*_O_*y*_ is formed from subcycles of Li_2_O + H_2_O and SiO_2_ + H_2_O, and its Li content can be conveniently controlled by the ratio of these subcycles.^102^

The prototypical role of aluminum oxide ALD based on the precursors trimethylaluminum (TMA) and H_2_O, together with the high diffusivity of the small Li^+^-ions, have formed a natural basis for a number of studies aiming at different Li–Al–O thin films. An example is the ternary process combining subcycles of LiO^*t*^Bu + H_2_O and TMA + H_2_O.^15,92^ In one study, stable growth was observed after 20–30 ALD cycles only when the proportion of the LiO^*t*^Bu + H_2_O subcycles was ≤50% and the Li/Al ratio ≤55%. For thinner films, a Li/Al ratio of ≤82% was achieved with a higher proportion of LiO^*t*^Bu + H_2_O subcycles.^15^ In variations of these processes, O_3_ has been used instead of H_2_O for the Al-based subcycle,^89,90^ and in addition to this, Li-TMSO as the Li precursor in place of LiO^*t*^Bu.^48,93^ In another ternary Li–Al process, LiAlF_4_ films were deposited, as an alternative to the LiF and AlF_3_ protective cathode coatings, through subcycles of LiO^*t*^Bu + TiF_4_ and AlCl_3_ + TiF_4_.^69^ Also, amorphous Li–Al–S films with the measured Li/Al ratio of 2.9–3.5 have been fabricated for use as a solid-state electrolyte by combining the processes for Li_2_S (LiO^*t*^Bu + H_2_S) and Al_2_S_3_ (Al(NMe_2_)_3_ + H_2_S).^94^

Besides aluminum, ternary Li–M–O thin films have been deposited for several other metal constituents: Ti,^107,108^ Mn,^97,99^ Co,^95,96^ Nb,^16,100^ Ta,^103−106^ and Sn,^120^ by using various three-precursor processes. In particular, Li_*x*_TiO_*y*_, Li_*x*_MnO_*y*_, and Li_*x*_CoO_*y*_ are all important electrode materials in LIBs. For Li_*x*_TiO_*y*_, depositions from LiO^*t*^Bu, Ti(O^*i*^Pr)_4_ (O^*i*^Pr = tetraisopropoxide) and H_2_O at 225 °C yielded films with high Li concentrations that could be further controlled by varying the Li and Ti precursor pulsing ratio. These films had improved stability in air compared to the more hygroscopic Li_*x*_TiO_*y*_ films deposited using TiCl_4_ as the Ti source.^107^ Ti(NMe_2_)_4_ ((NMe_2_)_4_ = tetrakis (dimethylamino); TDMA) has also been used to deposit Li_*x*_TiO_*y*_; in this case, the process did not require the H_2_O pulse between the two metal precursors.^108^ Both processes resulted in a spinel Li_4_Ti_5_O_12_ structure after annealing,^108^ or even as-deposited.^107^ For Li_*x*_MnO_*y*_ depositions, various precursor combinations have been tested at 225 °C: Li-THD, Li-HMDS, and LiO^*t*^Bu combined with Mn-THD and bis(ethylcyclopentadienyl)-Mn(II) (Mn(EtCp)_2_); as the oxygen source, O_3_ was used with THD-based precursors and H_2_O with the others.^97,99^ It was found that the Li precursor tends to react not just on the surface layer but also somewhat deeper,^97^ and in a follow-up study, this intercalation of Li^+^ into MnO_2_ films was investigated in more detail.^99^ For the Co-containing Li_*x*_CoO_*y*_ films, deposition experiments were carried out at 325 °C in a remote plasma process using LiO^*t*^Bu, CoCp_2_ (Cp = bis-cyclopentadienyl) and ^P^O_2_ as precursors; the Li/Co ratio could again be controlled by adjusting the precursor ratio.^95,96^

The Nb- and Ta-based compounds, LiNbO_3_ and LiTaO_3_, are interesting ferroelectrics. However, in the context of ALD, they have been mainly investigated as solid-electrolyte materials. Crystalline LiNbO_3_ films were deposited using Nb(OEt)_5_ as the Nb precursor.^16,100^ In the first study, attempts using the 1:1 ratio of the Li-HMDS and Nb(OEt)_5_ precursors resulted in uncontrolled growth, presumably due to the migration of Li^+^ ions onto the film surface. This could be mitigated by depositing a thick Nb_2_O_5_ layer (2000 cycles) in between.^16^ However, in a later study, linear growth was achieved even with the 1:1 ratio, possibly owing to the different Li precursor in LiO^*t*^Bu, and the longer purge times used. For the Li–Ta–O system, amorphous films were obtained at 225 °C from LiO^*t*^Bu, Ta(OEt)_5_ and H_2_O, but according to X-ray photoelectron spectroscopy (XPS) and X-ray absorption near edge structure data, the chemical environment in the films was very similar to that of LiTaO_3_.^104^ Like with other ternary processes, the Li/Ta ratio could be controlled by the precursor pulsing ratio.^103^

Lithium tin oxide films have been investigated as promising candidates for a negative electrode material. These films were deposited from Li-HMDS, SnEt_4_, and different oxygen coreactants, but were found to contain significant C and Si impurities (resulting from the use of Li-HMDS), even to the point that these could be described as core constituents.^120^ Indeed, some ternary ALD processes of Li-containing material can yield quaternary products, such as LiB_*x*_C_*y*_O_*z*_^111^ and LiNi_*x*_Si_*y*_O_*z*_;^115^ both oxygen sources investigated (O_2_-plasma and O_3_) were found to decompose the metal precursors such that the different elements effectively incorporated into the films. The degree of carbon incorporation could be adjusted by changing the deposition temperature, with lower temperatures resulting in higher C content.^111^

### Quaternary Li-Based Processes

The four-precursor ALD processes for Li-based thin films have mostly been developed with an eye on prospective solid-state electrolyte applications, and they have involved Al and Si,^61,109^ La and Ti,^9,113^ La and Zr,^114^ as well as Ti and P^118^ as the other two metal/cation constituents, and oxygen as the counter negative ion. The films have typically been deposited using LiO^*t*^Bu as the Li source and H_2_O or O_3_ as the oxygen source, depending on the reactivity of the metal precursor preceding it in the ALD cycle.

Lithium aluminosilicate thin films have been fabricated from LiO^*t*^Bu, TMA, and Si(OEt)_4_ precursors using H_2_O as the coreactant. In one study, the process was designed such that it started with the TMA + H_2_O subcycle (due to the well-known adhesion of TMA to silicon), and the films were grown at 290 °C by pulsing the precursors in a sequence of TMA, LiO^*t*^Bu, and Si(OEt)_4_ (each followed by a H_2_O pulse) onto Si and Ge substrates as well as onto Si nanowires. It was noticed that, at the chosen deposition temperature, silicon oxide layers did not grow alone but only as a constituent of the complex oxide. The films were deemed pinhole-free via electrochemical measurements and conformal following SEM imaging of the coated nanowires.^109^ Another group deposited Li–Al–Si–O films at 225 °C and investigated the surface reaction mechanism with FTIR, finding that the process proceeds along the ideal ALD chemistry lines, incorporating SiO_2_ into the complex oxide.^61^

Lithium lanthanum titanate thin films were reported already in the first Li ALD publication.^9^ The following subcycles were used at 225 °C: LiO^*t*^Bu + H_2_O, La-(THD)_3_ + O_3_ and TiCl_4_ + H_2_O. The carbon contamination level was determined to be 2–3 atom % with time-of-flight elastic recoil detection analysis (TOF-ERDA); this was considered low and ascribed to the use of LiO^*t*^Bu + H_2_O cycles instead of, for example, Li-THD + O_3_ for lithium incorporation.^9^ In the follow-up work, which involved more detailed TOF-ERDA measurements, the Li and La contents were found essentially equal, but Ti was seen to be in excess in the films. The former was deemed positive, considering a Li:La ratio close to unity typically yields the highest ionic conductivities for LiLa_*x*_Ti_*y*_O_*z*_ thin films. Additionally, secondary-ion mass spectrometry analysis found a mostly uniform film composition.^113^

Another Li- and La-containing material, lithium lanthanum zirconate (Li_7_La_3_Zr_2_O_12_), was grown from LiO^*t*^Bu, La-FAMD (FAMD = tris(*N,N*-di-isopropylformamidinato)), and Zr-TDMA; ozone was selected as the oxygen source for all subcycles to avoid issues arising from the hygroscopicity of the films and their binary components. The films were also doped with Al from TMA. To reach the cubic Li_7_La_3_Zr_2_O_12_ phase with higher ionic conductivity, postdeposition annealing was required, but at a lower temperature of 555 °C compared to bulk Li_7_La_3_Zr_2_O_12_. The films were deposited onto single-crystal MgO(100) substrates to avoid unwanted film–substrate reactions during the annealing.^114^

Quaternary processes aiming at different phosphates of Li with Fe^112^ and Ti^118^ have been investigated, as these compounds are promising anode materials. For LiFePO_4_, the process consisted of LiO^*t*^Bu + H_2_O, FeCp_2_ (ferrocene) + O_3_ and TMP + H_2_O subcycles at 300 °C. The growth was shown to be linear on Si substrates and conformal on CNTs. The as-deposited films were amorphous, and they crystallized upon annealing at 700 °C (for 5 h) into orthorhombic LiFePO_4_.^112^ For the Li–Ti–P–O system, nanocomposite films (consisting of anatase TiO_2_ crystals embedded in an amorphous lithium phosphate matrix) were deposited on Si and CNTs at 250 °C from LiO^*t*^Bu, TMP, and Ti(O^*i*^Pr) precursors. The growth was linear and yielded uniform and conformal coatings on the CNTs.^118^

### LiPO_4_ and LiPON Processes

The ALD processes for the three- and four-element LiPO_4_ and LiP_*x*_O_*y*_N_*z*_ (LiPON) thin films deserve special attention for two reasons: (i) These materials are very promising solid-electrolyte material candidates for thin-film Li-ion microbattery applications, and (ii) there are various different ALD approaches, regarding the number of precursors used, for incorporating the multiple elements into the film.

Lithium phosphate films have been successfully deposited through a two-precursor process from LiO^*t*^Bu and trimethyl phosphate (TMP). Depositions at 250–325 °C yielded amorphous or slightly crystalline films at a rate of ∼0.7 Å/c. The films were unstable upon long-term storage in ambient air and contained some carbon impurities which were lesser at lower temperatures.^81^ At higher deposition temperatures, better ionic conductivities were achieved for the films.^82^ The films were moreover shown to be conformal even on high-aspect-ratio surfaces.^83^ In later experiments, an additional H_2_O pulse was included into the LiO^*t*^Bu + TMP process to help the TMP react fully with the LiO^*t*^Bu-terminated surface. This was found in turn to considerably reduce the carbon incorporation into the film, even below 1%.^85^ The same process was later utilized to deposit nearly amorphous Li_3_PO_4_ films at 200 °C.^86,87^ Though these works were primarily conducted in the context of LiPON research, the authors demonstrated the growth of non-nitrogen-doped Li_3_PO_4_ (along with Li_2_CO_3_) inside a mesoporous oxide to produce nanocomposite electrodes.^64^

For LiPON, both binary two-precursor and quaternary four-precursor processes have been successfully developed.^52−54^ In the two-precursor processes, diethyl phosphoramide (DEPA; PO(NH_2_)(OEt)_2_) works as a simultaneous source for P, O, and N, which can then be combined with LiO^*t*^Bu or Li-HMDS as a lithium precursor.^81−83^ Here, the advantage of Li-HMDS is its stability in ambient air. The particular advantage of DEPA is that it contains the P–N bonds required for LiPON; these bonds are otherwise difficult to incorporate into the film using conventional phosphorus and nitrogen ALD precursors.^52^ The growth rate of the LiPON process seemed to increase with deposition temperature, from 0.15 Å/c to 0.9 Å/c at 200–300 °C, in line with a thermally activated reaction.^53^ Stoichiometries were observed at Li_0.95_PO_3_N_0.6_^52^ and close to Li_2_PO_2_N.^53^

The four-precursor processes for LiPON differ from the other quaternary processes in that the oxygen coreactant is pulsed only once per overall cycle, and instead a nitrogen source, NH_3_ or N_2_-plasma (^P^N_2_) is introduced. As the Li precursor, LiO^*t*^Bu has been employed in all works, and phosphorus has been supplied via TMP or P(NMe_2_)_3_ (TDMAP). In several studies, the same plasma-based process of LiO^*t*^Bu, H_2_O, TMP, and ^P^N_2_ at 200–275 °C has been investigated,^85,86,116^ while one study focused on a thermal-ALD process using LiO^*t*^Bu, NH_3_, TDMAP, and O_2_ at a high deposition temperature of 400–500 °C; the thermal-ALD process yielded carbon-free and highly conformal LiPON coatings on LiCoO_2_ substrates.^117^ For the plasma processes, it was found that varying the ^P^N_2_ dose enabled the tuning of the nitrogen content, such that the films with nitrogen content higher than 4.5% were amorphous, while otherwise crystalline.^85^ A later study from the same group focused on the electrochemical properties of LiPON as an electrode coating.^116^ Another group deposited LiPON as part of a solid-state battery.^86^ All LiPON processes have been found to be moderately or highly conformal.

## 5 Other Alkali Metal-Based ALD Processes

Comparatively little work has been carried out for alkali metals other than lithium; these ALD processes are summarized in Table 2. The pioneering paper is from the year 2014, five years after the first Li ALD publication. In this work, Østreng et al.^49^ explored the deposition of oxygen-based sodium and potassium compounds (aluminates and silicates), with a focus on the properties of the Na and K precursors. The work was partly motivated by prospective superconductor, thermoelectric, dielectric, and piezoelectric applications. Three different precursor types were investigated: *tert*-butoxides (MO^*t*^Bu), trimethylsilanolates (M-TMSO), and hexamethyldisilazides (M-HMDS). Of these, MO^*t*^Bu and Na-TMSO were found to facilitate ALD-like growth. In later studies, the O^*t*^Bu precursors have been exclusively used, and Na- and K-containing films have often been studied in the same works.

**Table 2:** Na-, K-, Rb-, and Cs-Containing Thin Films Deposited with ALD^a^

material	precursor 1	precursor 2	precursor 3	precursor 4	GPC (Å/c)	*T* (°C)	ref
NaO_*x*_	Na-THD	O_3_			0.2	–	(121)
NaF	NaO^*t*^Bu	HF(-pyridine)			0.9	175–200	(122)
NaSi_*x*_O_*y*_	Na-TMSO	O_3_			0.7–1.8	250–350	(49)
NaAl_*x*_O_*y*_	NaO^*t*^Bu	AlMe_3_	O_3_		–	240	(88)
	NaO^*t*^Bu	AlMe_3_	H_2_O^a^/O_3_^a^		2.9–3.2	225–375	(49)
	Na-TMSO				–		
NaCo_*x*_O_*y*_	Na-THD	Co(acac)	O_3_^a^		0.2–0.3	220–250	(121)
NaNb_*z*_O_*y*_	NaO^*t*^Bu	Nb(OEt)_5_	H_2_O^a^		1.3	200–300	(123)
NaNb_*z*_Ta_*y*_O_*z*_	NaO^*t*^Bu	Nb(OEt)_5_	Ta(OEt)_5_	H_2_O^a^	–	250	(124)
NaTa_*z*_O_*y*_	NaO^*t*^Bu	Ta(OEt)_5_	H_2_O^a^		1.3	200–300	(123)
Na_4_PO_3_N	NaO^*t*^Bu	PO(NH_2_)(OEt)_2_			1.0	375–400	(125)
KNa_*x*_Nb_*y*_O_*z*_	KO^*t*^Bu	NaO^*t*^Bu	Nb(OEt)_5_	H_2_O^a^	–	250	(123, 126)
KNa_*x*_Ta_*y*_O_*z*_	KO^*t*^Bu	NaO^*t*^Bu	Nb(OEt)_5_	H_2_O^a^	–	250	(123)
KAl_*x*_O_*y*_	KO^*t*^Bu	AlMe_3_	H_2_O^a^		0.9–1.3	250	(49)
KNb_*x*_O_*y*_	KO^*t*^Bu	Nb(OEt)_5_	H_2_O^a^		1.1	200–300	(51, 123)
KTa_*x*_O_*y*_	KO^*t*^Bu	Ta(OEt)_5_	H_2_O^a^		1.2	200–300	(123)
KNb_*x*_Ta_*y*_O_*z*_	KO^*t*^Bu	Nb(OEt)_5_	Ta(OEt)_5_	H_2_O^a^	–	250	(123)
RbNb_*x*_O_*y*_	RbO^*t*^Bu	Nb(OEt)_5_	H_2_O^a^		0.6–0.7	250	(46)
RbTi_*x*_O_*y*_	RbO^*t*^Bu	Ti(O^*i*^Pr)_4_	H_2_O^a^		0.5–0.6	250	(46)
Cs_*x*_NbO_*y*_	CsO^*t*^Bu	Nb(OEt)_5_			–	–	(30)

a Denotes an O precursor pulsed after every non-O precursor.

### Na- and K-Based Processes

**Figure 6:**
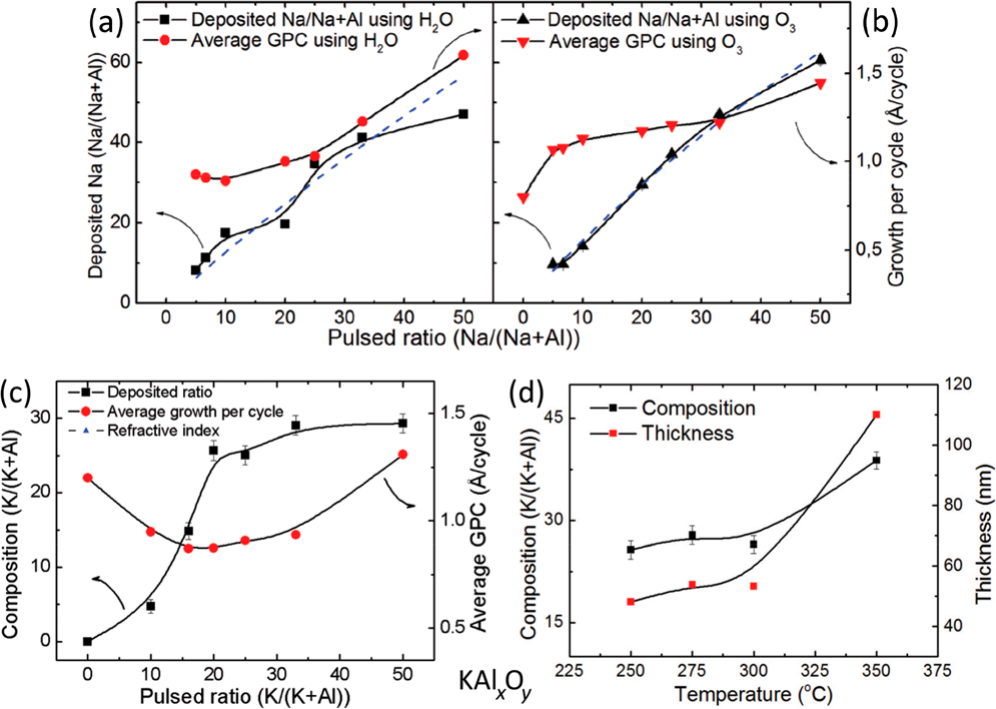
Growth characteristics and resulting film composition for: (a, b) NaAl_*x*_O_*y*_ films with different Na/Al ratios (inset: refractive indices); (c) KAl_*x*_O_*y*_ films different K/Al ratios; and (d) thickness and composition of KAl_*x*_O_*y*_ films deposited at different temperatures. Adapted with permission from ref (49). Copyright 2014 Royal Society of Chemistry.

The first ALD experiments based on Na and K were for aluminates and silicates, as the ALD of pure oxides or hydroxides was deemed problematic (due to CO_2_ and H_2_O intake during/after the deposition). NaO^*t*^Bu and Na-TMSO were used to deposit NaAl_*x*_O_*y*_ and NaSi_*x*_O_*y*_ films at 225–375 °C. H_2_O was used as the oxygen source in all of these processes, except for one NaAl_*x*_O_*y*_ process that employed O_3_. NaO^*t*^Bu was tested at temperatures of 125–150 °C, whereupon the process reached a growth rate plateau for ≥140 °C. Similarly, KO^*t*^Bu, TMA, and H_2_O were used to deposit KAl_*x*_O_*y*_ films at 300 °C. In Figure 6, the growth characteristics and the resultant film compositions for these processes are presented as a function of the precursor pulsing ratios. The films were amorphous, and uniformity was achieved with longer pulse times and when the K:Al deposition ratio was kept relatively low (1:4). As discussed in Section 3, other Na and K precursors (Na-HMDS, K-HMDS, K-TMSO) were examined too, but soon deemed unsuitable for ALD, as they seemed to decompose instead of sublimating when heated.^49^

In follow-up work from the same group, perovskite-type A^I^B^V^O_3_ (A = K, Na; B = Nb, Ta) films were grown at 200–300 °C from NaO^*t*^Bu, KO^*t*^Bu, Nb(OEt)_5_, and Ta(OEt)_5_. Each metal precursor pulse was followed by an H_2_O pulse. The ratio of A to B was altered by varying the relative number of pulses for each precursor. Pulsing ratios between 1:1 and 1:9 yielded reproducible films, in which even the 1:9 ratio still resulted in considerable amounts (20–30%) of A, likely due to the alkali metals diffusing deeper than the surface of the film during the deposition. For these lower A ratios, the resultant alkali-metal content in the films was slightly higher for K than for Na, presumably due to differences between the alkali precursor oligomers. The ternary K films also had reduced growth rates at higher ratios, while ternary Na films did not. At higher deposition temperatures (275–400 °C), the K- and Na-based films had opposite growth rate trends, rising and falling, respectively. Here, an increasing growth rate correlated with a decreasing A:B ratio, and vice versa.^123^

The deposition of KNbO_3_ was explored further in another study that focused on the use of KO^*t*^Bu. The results suggested that KO^*t*^Bu tetramers chemisorb to the sample surface and deposit excess KOH upon the consequent H_2_O pulse. The volatile KOH then further reacts with the next pulsed precursor, Nb(OEt)_5_ and is purged from the surface. The KNbO_3_ species are hygroscopic, which in turn increasingly affected the film growth with an increasing number of deposition cycles.^51^

The ALD process development for K(Ta,Nb)O_3_ films was motivated due to its promising electro-optical properties; this material could also serve as a Pb-free replacement component for piezo- and ferroelectric devices. The K concentration of the quaternary oxide affected the phase formation, and the orientation was controlled by the substrate choice; particularly good results were obtained using substrates with similar lattice constants to the material.^124^ Chemically and structurally uniform deposition of the material was possible with a high degree of control over its elemental constituent concentrations.^126^

More recently, Na- and K-based films have also been deposited using additional constituents other than Al, Nb, or Ta. For example, conformal films of the LiPON analogue of sodium, Na_4_PO_3_N (NaPON), were grown at 300–375 °C from NaO^*t*^Bu and DEPA precursors. The process appeared nonideal, though: The growth rate increased in tandem with deposition temperature, including a steep increase from 0.2 Å/c at 325 °C to 1 Å/c at 375 °C. Some incorporation of carbon was detected, likely due to precursor decomposition at the high deposition temperatures.^97,125^ Additionally, NaF films have been deposited with HF-pyridine as the fluorine precursor. At 175–200 °C, 0.85 Å/c growth was achieved, and the roughness of thin (<10 nm) NaF films was reasonably low despite them being crystalline.^122^

In another study, thermoelectric Na_*x*_CoO_2_ films were aimed at; the films were grown using Na-THD as the sodium source and O_3_ as the oxygen source. Instead of continuous growth, the films exhibited a pattern of separated channels 10–20 μm wide and over 100 nm high for the nominal Na content varying between *x* = 0.33–2.0. This growth pattern was in contrast to the smooth films previously obtained for Co_3_O_4_ (*x* = 0).^121^

### Rb- and Cs-Based Processes

The similarities in alkali-metal ALD processes have been further studied with the heavier alkali metals, Rb^46^ and Cs.^30^ Their precursors were not commercially available and were thus synthesized in-house, with procedures similar to those for other alkali metal MO^*t*^Bu precursors. These precursor compounds were moisture-sensitive white solids, with hygroscopic and ionic behavior increasing with atomic weight. The resulting films also tended to react with ambient humidity.^30^

Due to the earlier-seen difficulties in depositing binary alkali metal oxide films, the ALD of Rb-based films was started by aiming at ternary oxides with Ti and Nb from RbO^*t*^Bu, Ti(O^*i*^Pr)_4_, and Nb(OEt)_5_; the oxygen precursor was H_2_O. The Rb content of the films could be tuned up to a Rb:TiO_*x*_ ratio of 1:4, similarly to the case of Na and K. The use of Nb allowed for higher Rb contents, including the 1:1 composition of RbNbO_3_ on a SrTiO_3_ substrate after annealing. This was noted as a strength of ALD, as the complex high-Rb-content structure was unattainable via conventional (physical) deposition methods.^46^

Deposition of Cs-containing films was attempted as part of an expansive O^*t*^Bu-precursor study.^30^ Very similar growth characteristics to the earlier KO^*t*^Bu and RbO^*t*^Bu depositions were seen, with a high Cs intake of >50% observed with a mere 1:5 Cs:Nb precursor pulse ratio. Complex oxides capable of stabilizing CsO_*x*_ or CsOH proved difficult to find, but it was possible to employ ALD in inserting Cs into NbO_*x*_ films. A well-behaving ALD process for Cs would be desired to realize, for example, CsPbI_3_ films for solar cells.^30^

## 6 Alkali Metal-Based ALD/MLD Processes

The first ALD/MLD process involving alkali metals was a process for lithium terephthalate films reported in 2016.^23^ Since then, more than 20 processes have been reported, as listed in Table 3. These metal–organic materials comprise, besides Li–organics, other alkali metal compounds as well, where the alkali-metal nodes are linked together with organic linkers.^22,137^

**Table 3:** ALD/MLD Processes for Alkali-Metal-Containing Metal–Organic Films^a^

material	precursor 1	precursor 2+	GPC (Å/c)	*T* (°C)	crystallinity	ref
Li-EG	Li-HMDS	EG	2.5–3	80	amorphous	(127)
	LiO^*t*^Bu	EG	2.6	135–150	amorphous	(128, 129)
Li-PD	LiO^*t*^Bu	1,3-PD	0.23	140–200	substrate-dependent	(130)
Li-EG-CO_2_	Li-HMDS	EG + CO_2_	2.5–3	80	amorphous	(127)
Li-GL	LiO^*t*^Bu	GL	27	150	amorphous	(129)
Li-HQ	Li-HMDS	HQ	4.5	105–280	crystalline	(25, 54)
	LiO^*t*^Bu	HQ	–	150	crystalline	(129)
Li-BDS	Li-THD	H-BDS	2.0–2.3	200–260	crystalline	(131)
Li-TPA	Li-THD	TPA	3	200–280	crystalline	(14, 23, 132, 133)
Li-ATPA	Li-THD	2-ATPA	3.6	200	crystalline	(132)
Li-PDC	Li-THD	3,5-PDC	2.5	190–300	crystalline	(24, 133)
Li-NDC	Li-THD	2,6-NDC	2.3	220	crystalline	(133)
Li-BPDC	Li-THD	4,4′-BPDC	7	240	crystalline	(133)
Li-AZO	Li-THD	4,4′-AZO	7	270	crystalline	(133, 134)
Na-TPA	Na-THD	TPA	3	190–300	crystalline	(14)
Na-PDC	Na-THD	3,5-PDC	3.7	190–300	crystalline	(24)
Na-adenine	Na-THD	adenine	∼10	260–320	crystalline	(47)
Na-uracil	Na-THD	uracil	4.8	260–320	crystalline	(47, 135, 136)
K-TPA	K-THD	TPA	2.5	190–300	crystalline	(14)
K-PDC	K-THD	3,5-PDC	3.5	190–300	crystalline	(24)

a For the precursor abbreviations, see Figure 8.

**Figure 7:**
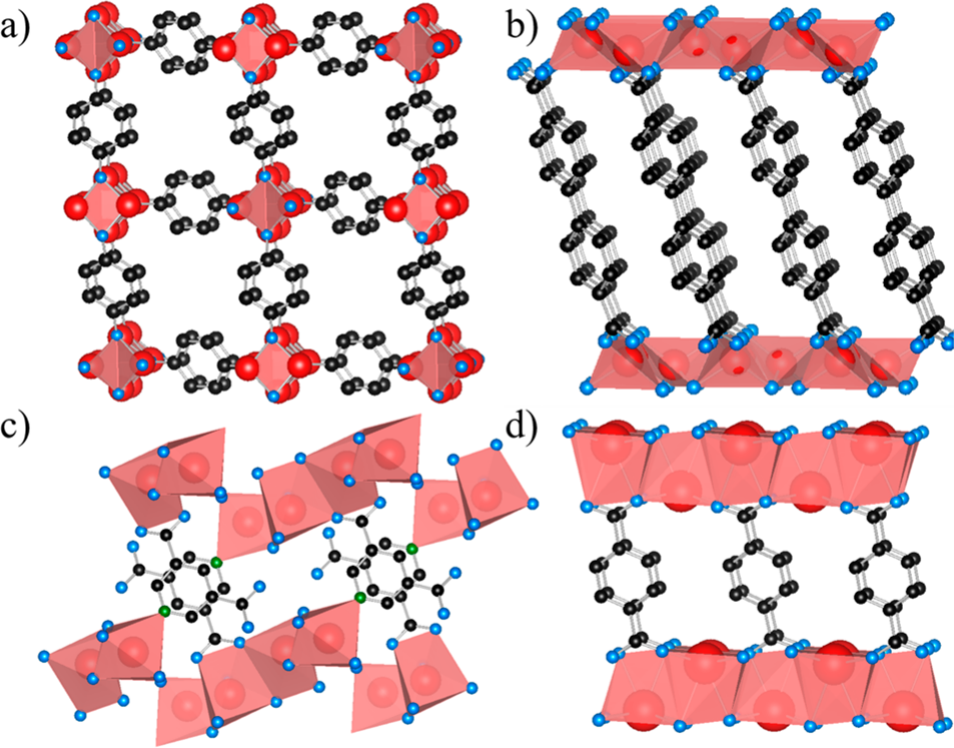
Crystal structures of representative ALD/MLD-fabricated alkali metal–organic materials drawn with Vesta:^144^ (a) Li-HQ,^25^ (b) Li-4,4′-BPDC,^145^ (c) Na-3,5-PDC,^146^ and (d) K-TPA;^147^ (b and d) have a layered structure, (c) has a 3D structure and involves pyridyl nitrogen, and (a) has unsaturated Li sites.

Many of the alkali metal–organic thin films grow in situ crystalline.^14,23,24,132,133^ As a plausible explanation, it is believed that the crystallinity is promoted by the nondirectional ionic bonds that allow a stress-free structure to form during the deposition. This fact provides us with attractive opportunities to fabricate coordination polymer (CP) or MOF-like materials in high-quality thin-film form. Examples of these ALD/MLD-grown alkali-metal-based metal–organic compounds/crystal structures are shown in Figure 7. An exciting fact is that many of these compounds/structures are fundamentally new: they have not been realized through any other synthesis route so far.

**Figure 8:**
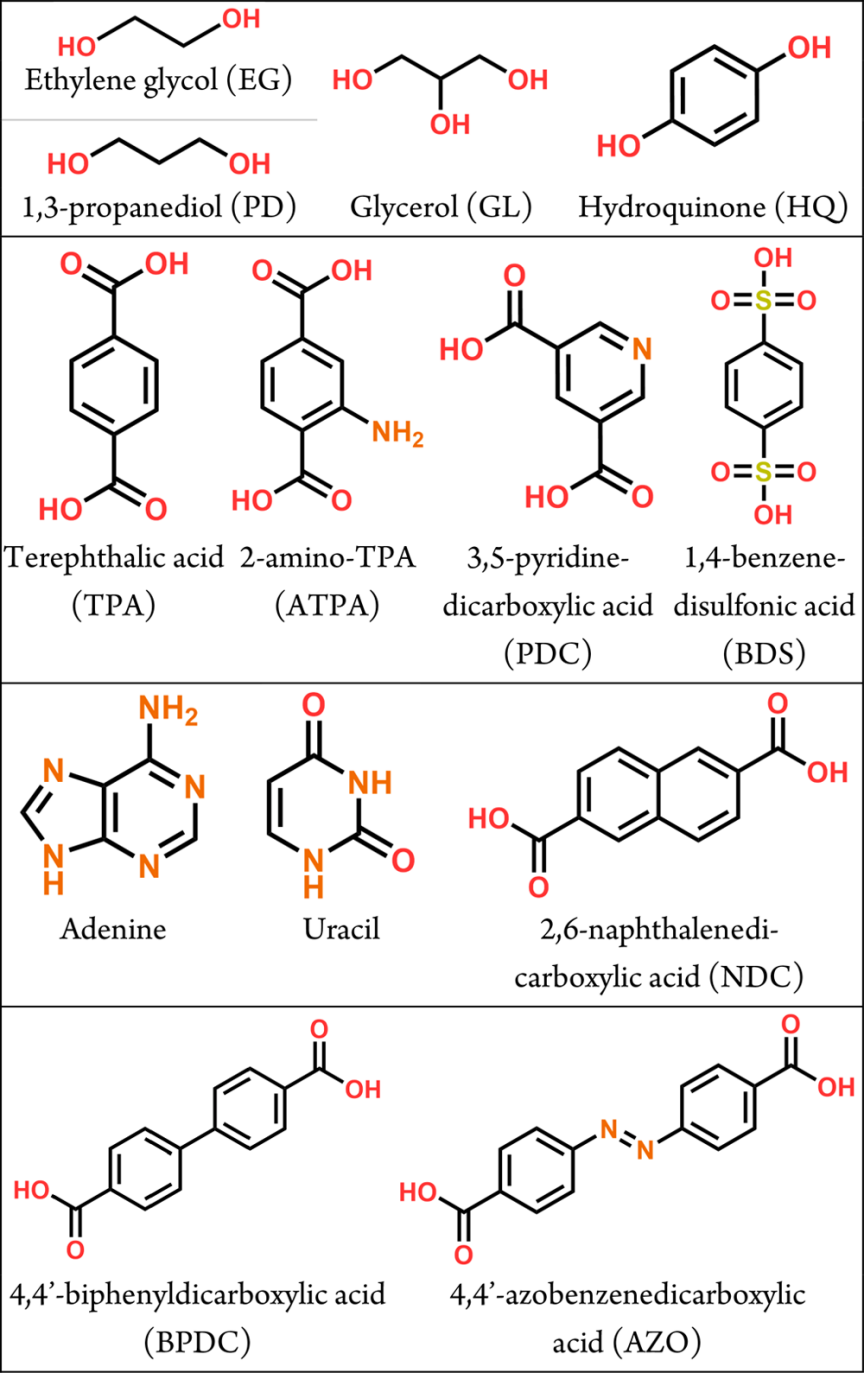
Organic precursors used in connection with alkali metals in ALD/MLD.

In the ALD/MLD processes, THD-based precursors have been most commonly employed for the alkali metals. This choice is motivated by their stability as well as their sublimation temperatures, which match quite well with those of the commonly employed organic precursors. For the use of the (larger) organic precursors (Figure 8), some extra considerations are needed, as the organic molecules typically suffer from low vapor pressures. Accordingly, there can be a significant delay in saturation of the reactions depending on where the substrate is positioned inside the reactor. If a metal precursor with small ligands is used, the pulse time of the organic precursor must be increased to counteract the steric hindrance.^138^ Large organic molecules may thermally decompose before they sublime, even under a reduced pressure.

Organic molecules can also be sticky, which means that they may be also incorporated in the film in a non-ALD/MLD fashion. This could happen for the metal precursors as well, as they may diffuse inside the film, causing deviations from the ideal behavior.^19,137,139−143^ Such behavior is often seen as a large variation in the GPC vs deposition temperature graph. However, even if this occurs, the process may still be well-controlled in an ALD/MLD manner, showing saturation of the surface reactions, good uniformity, and predictable growth rates. This should not be an unsolvable obstacle for industrial applications either.^45^

By far the most common metal in alkali ALD/MLD films is lithium, which has been combined both with aliphatic and aromatic organic molecules, resulting in films with various properties and also different stabilities. The preferred coordination number of lithium is 4, and this requirement is fulfilled with aromatic dicarboxylates and pyridine dicarboxylates, resulting in very stable crystalline structures. On the other hand, lithium alkoxides (coordination number 2) are very sensitive to moisture or even CO_2_.

### Aliphatic Li–Organic Films

The first reported ALD/MLD process for aliphatic Li–organic films was that for Li-1,3-PD (Li-PD) by Wang et al.^130^ in 2020, shortly followed by us for Li-EG and Li-EG-CO_3_ films.^127^ An ALD/MLD process for Li-EG was also realized using LiO^*t*^Bu, and its Li^+^ ionic conductivity was measured for the first time.^128^ The Li-EG films were amorphous with a very similar growth profile (maximum GPC ∼ 2.6 Å/c) and morphology regardless of the precursor used.^127,128^ The Li-PD films were crystalline when deposited on a crystalline substrate and showed lower growth rates (0.8 Å/c at 100 °C), which was attributed to double reactions of the PD precursor inhibiting further reactions.^130^ All the alkoxide processes showed significant nucleation delay and island-type growth.

The decomposition of the lithium alkoxide in Li-PD films (to Li_2_CO_3_) happens via an alkyl carbonate intermediate.^130^ The lithium alkyl carbonates are more stable than lithium alkoxides, and we pioneered an ALD/MLD process using CO_2_ as a reactant in the Li-EG process to directly deposit films of lithium hydroxyethylene carbonates (Li-EG-CO_3_).

The formation of alkyl carbonate is evident from the strong IR vibrations bands of carbonate at 1700–1300 and 825 cm^–1^. These films were also amorphous, but interestingly, the initial nucleation delay vanished, and the roughness of the films decreased significantly, highlighting the significant effects that the CO_2_ had on this process. Using CO_2_ is not limited only to Li-EG, as other aliphatic lithium alkoxides and even other group 1 alkoxides should undergo similar reactions.

Very recently, a Li-GL process was developed for coating Li-metal anodes.^129^ According to SEM image analysis, it yielded amorphous films at a very high average growth rate of ∼27 Å/c, much higher than any alkali-metal-based ALD/MLD process reported to date. From QCM measurements, a far higher mass gain per cycle was revealed for Li-GL than for similarly grown Li-EG and Li-HQ films. Lithium content in the films varied by film thickness (20–50 atom %), increasing toward the film–substrate interface, according to XPS.

### Aromatic Li–Organic Films

The study of Li–organic films started with the deposition of dilithium terephthalate (Li-TPA), which is an interesting organic electrode anode material.^23^ The films were very stable in atmospheric conditions due to the filled coordination of lithium. Other lithium compounds that are coordinated with carboxylate have also been shown to be very stable with no hydrate formation.^24,132,133^ The deposited films are crystalline, with a similar crystal structure to the bulk phase. The deposition of lithium carboxylates has only been reported with Li-THD, largely at temperatures over 200 °C. The temperature is intrinsically limited by the source temperatures of the carboxylic acid organic precursors. The GPC in all the processes somewhat depended on the organic backbone length and decreased rapidly with increasing depositing temperature in all the lithium carboxylate ALD/MLD processes.^133^

Crystalline lithium aryloxide films were also deposited from hydroquinone and Li-HMDS.^54^ The process showed typical ALD/MLD behavior with strong temperature dependence. The films were highly sensitive to ambient moisture, which transformed the crystal structure. The films could be dried, retaining the initial crystal structure. The film could also be protected by depositing a thin ALD Al_2_O_3_ coating, allowing easy characterization of the film underneath. The gas-phase deposition route provided by ALD/MLD to manufacture crystalline MOF-like structures without intercalated solvent molecules is a very strong motive to continue the study of these materials.

### Other Alkali Metal–Organic Films

The ALD/MLD growth of Na-TPA and K-TPA films is in general very similar to that of Li-TPA, so there seem not to be huge differences between the alkali metal constituents.^14^ Changing the organic component, however, does affect the water absorption behavior. The crystal structure of the Na-PDC film matches well with the reported bulk structure, with similar water absorption behavior. The K-PDC films were hygroscopic and a thin Al_2_O_3_ coating was required to experiment with the nonhydrated phase. This is very different behavior from that of the Na-TPA and K-TPA films, as neither of those films absorbed water and both were very stable. The organic molecule alone can therefore have a large effect on the stability of the material.

Other ALD/MLD-grown alkali metal–organic films include the Na-adenine and Na-uracil films, which have potential applications in luminescence sensors and organic LEDs. Both films were crystalline, with a very high growth rate and no clear temperature dependence. The crystallinity and high GPC values were believed to be due to a smaller number of the (bulky) THD ligands in the case of the Na-THD precursor, compared to the Ba-(THD)_2_ and La-(THD)_3_ precursors investigated in the same study. This is an interesting observation, as the alkali-metal–PDC films compared to alkaline-earth-metal–PDC films showed this same behavior. Alkali-metal–PDC films were crystalline, while alkaline-earth-metal–PDC films were not, but the growth rate was very similar in both cases.^24^ The alkali- or alkaline-earth-metal–TPA analogues were all crystalline.^14^ All these studies were done with M-THD ligands, but to test this assumption with smaller ligands such as O^*t*^Bu, it could be deposited with, for example, PDC to see if it has any effect on the crystallinity of the films.

## 7 Functional Properties and Applications

**Figure 9:**
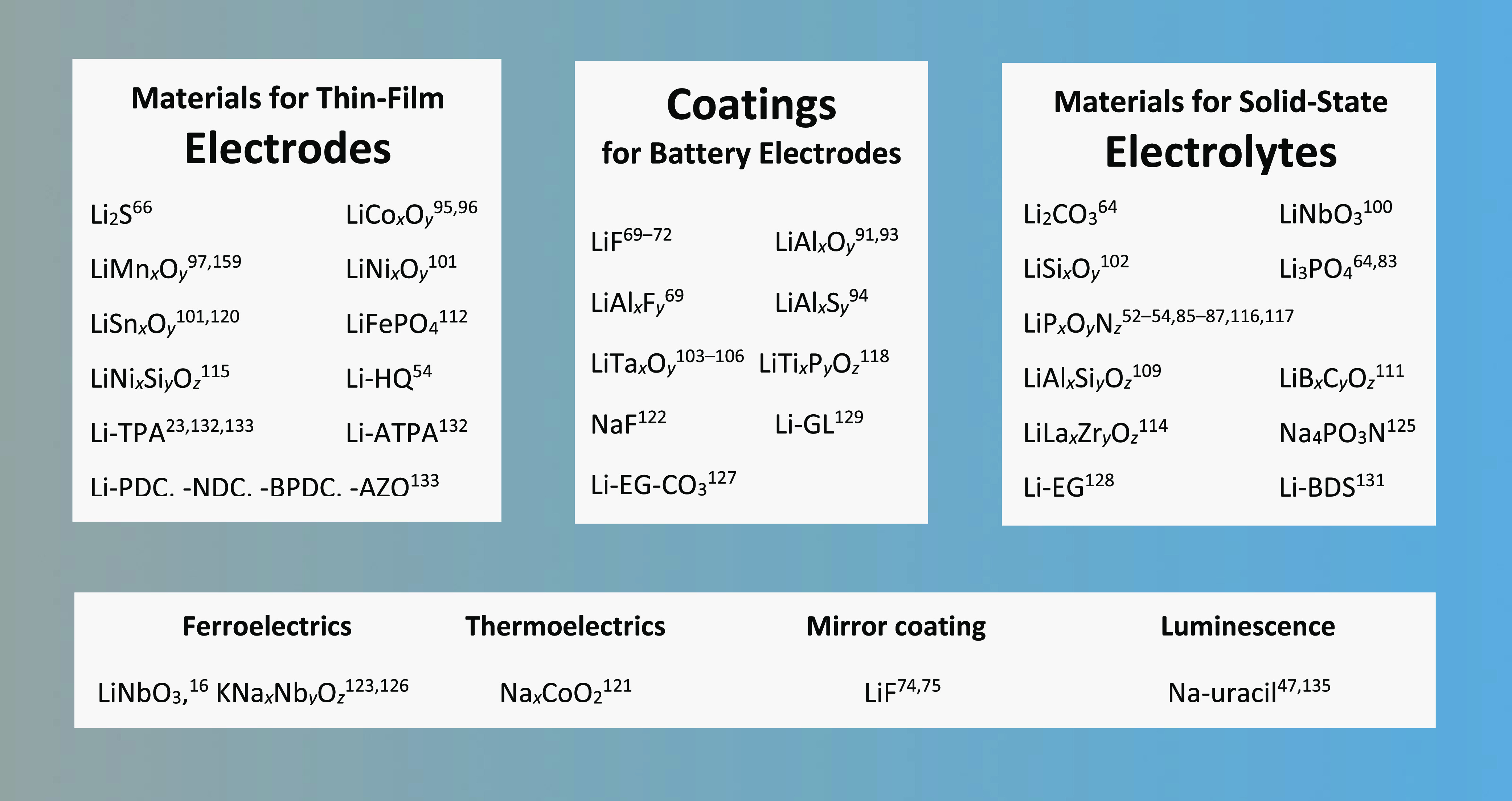
Future applications intended for ALD- and ALD/MLD-grown alkali-metal-based thin films.

The most commonly targeted application area for the alkali-metal-containing ALD and ALD/MLD films is in different electrochemical systems, typically as a solid-state electrolyte, but also as an active electrode material or an interface-coating layer. This is clearly seen from Figure 9, where we have collected the potential applications suggested for these films. The intended electrochemical performance is typically evaluated by measuring the ionic conductivity of the films. In Table 4, the reported (room temperature, RT) ionic conductivity values are listed. In this section, interesting examples of properties and applications of the alkali-metal-based ALD and ALD/MLD films are discussed, loosely following the order in which the materials were introduced in previous sections.

**Table 4:** Room Temperature (298–303 K) Ionic Conductivity Values Reported for ALD-Grown Alkali-Metal-Based Films^a^

material	ionic conductivity (S/cm)	ref
LiAl_*x*_O_*y*_	1.0 × 10^–10^–5.6 × 10^–8^	(91, 93)
LiAl_*x*_F_*y*_	3.5 × 10^–8^	(69)
LiAl_*x*_S_*y*_	2.5 × 10^–7^	(94)
LiNbO_3_	6.4 × 10^–8^	(100)
LiTa_*x*_O_*y*_	2.0 × 10^–8^	(104)
LiSi_*x*_O_*y*_	5.7 × 10^–9^	(102)
LiP_*x*_O_*y*_	3.3 × 10^–8^–4.3 × 10^–6^	(82−84)
LiP_*x*_O_*y*_N_*z*_	1.5 × 10^–7^–6.6 × 10^–7^	(52, 53, 85, 86, 117)
LiAl_*x*_Si_*y*_O_*z*_	1.0 × 10^–7^	(109, 110)
LiB_*x*_C_*y*_O_*z*_	2.2 × 10^–6^	(111)
LiLa_*x*_Zr_*y*_O_*z*_	1.0 × 10^–8^	(114)
NaP_*x*_O_*y*_N_*z*_	1.0 × 10^–7^	(125)
Li-EG	3.6 × 10^–8^	(128)

a If multiple values were reported (due to, e.g., varying material contents), the highest value in each publication was selected.

Ultrathin (20 ALD cycles) LiF and LiAlF_4_ films have been investigated as coatings for Li(Mn,Ni)O_2_ cathodes; while the LiF-coated cathodes suffered from reduced capacity due to the poor conductivity of LiF, a LiAlF_4_ coating improved the performance of the Li(Mn,Ni)O_2_ cathode.^69^ The choice of fluorine precursor may be of importance, as TiF_4_ was found to yield purer LiF, while Li-HFAC (hexafluoroacetylacetone) produced a porous LiF-CF_*x*_ hybrid with improved Li-ion conductivity.^71^ In addition to battery applications, ALD-grown LiF films have been considered for UV optics (e.g., UV windows),^67,74−77^ owing to the large optical band gap and low refractive index of LiF.^67^ However, deposition temperatures below 150 °C were found to result in films of lower microroughness and a higher refractive index, which could be beneficial when aiming at LiF coatings for aluminum mirrors.^77^

Lithium phosphate (Li_3_PO_4_) and especially its N-doped derivative (LiPON; disrupted PO_4_ chains and better Li-ion transport properties)^86^ are some of the most promising electrolyte materials grown by ALD. For the Li_3_PO_4_ films, the highest ionic conductivity values were achieved when using high deposition temperatures: For films deposited at 300 °C, 1.7 × 10^–7^ S/cm was measured at 50 °C by one group,^82^ and 6.2 × 10^–7^ S/cm at RT by another group.^83^ These values are orders of magnitude higher than that of crystalline bulk Li_3_PO_4_.^82^ Moreover, for these ultrathin (10 nm) films, appreciably high electrical resistance values could be simultaneously realized,^83^ which is another requirement for a solid-electrolyte material. For the LiPON films (deposited at 300 °C), RT ionic conductivity values as high as 6–7 × 10^–7^ S/cm were achieved.^52,53^ Most interestingly, the use of the thin LiPON films as a solid electrolyte in a practical microbattery in a combination with ALD/MLD-grown Li–organic electrodes could be demonstrated.^54^ For the sodium-counterpart NaPON, slightly lower ionic conductivity values were recorded: 1.0 × 10^–7^ S/cm at RT and up to 2.5 × 10^–6^ S/cm at 80 °C.^125^ Besides these phosphate materials, competitive ionic conductivity values have been reported for “LiB_*x*_C_*y*_O_*z*_” films (a mix of Li_3_BO_3_ and Li_2_CO_3_): up to 2.23 × 10^–6^ S/cm at RT, by controlling the Li_2_CO_3_ content.^111^ For the other electrolyte material candidates, the ionic conductivity values reached have been lower (see the RT values in Table 4).

In some studies, postdeposition annealing has been used for crystallization and subsequent enhancement of ionic conductivity. For postannealed crystalline LiAlSiO_4_ films, the ionic conductivity (1.3 × 10^–7^ S/cm) was 2 orders of magnitude higher than previously reported for amorphous LiAlSiO_4_ films.^110^ Similarly, annealing at 555 °C resulted in crystalline (cubic) Al-doped Li_7_La_3_Zr_2_O_12_ films with ionic conductivity values of 1.2 × 10^–6^ – 7.8 × 10^–5^ S/cm at 100–200 °C.^114^

Several Li-ALD processes have been utilized to fabricate electrode coatings. In a rather early example, ALD-Li_*x*_Ta_*y*_O_*z*_ coatings were used to improve the durability of Li(Ni,Mn,Co)O_2_ cathodes; the fact that the thicker coatings offered a better protection but also resulted in higher impedance underlines the necessity of optimizing the coating thickness.^105^ In another study, LiCoO_2_ electrodes were coated with an ALD-LiAlO_2_ film and cycled up to 50 times to demonstrate that the coated samples exhibited better capacity retention and retained largely the same charge transfer resistance, while the uncoated samples rapidly lost capacity and conductance.^92^ Also promisingly, LiAlF_4_ films have been found relatively stable when cycled at a wide electrochemical window of 2.75–4.50 V vs Li^+^/Li.^69^

Among the active electrode material candidates, ALD-Li_*x*_Mn_*y*_O_*z*_ films have exhibited promising galvanostatic cycling characteristics, remaining stable at approximately 200 mAh/g for 550 cycles at a voltage range of 2.2–4.5 V.^94^ In another study, LiFePO_4_ cathode films deposited onto CNTs and annealed at 700 °C for 5 h exhibited steady capacity retention at ca. 120 mAh/g even after 2000 charge–discharge cycles at a rate of 1 C;^112^ they also showed high electronic conductivity, even though the property appeared not to be intrinsic but rather due to the CNT network. Similarly, the TiO_2_/Li_3_PO_4_ nanocomposite films deposited on CNTs exhibited capacity retention at above 200 mAh/g for 200 cycles and good rate capability between 0.5C and 10C.^118^

With ALD/MLD, both electrode and coating materials have been realized.^23,28,51,127−132,148^ In particular, the Li–organic coatings could be beneficial as interfacial coatings or components for artificial SEI layers to be deposited on top of electrodes prior to the use. The argument here is that the stability of an electrode is improved by suppressing the harmful side reactions with the electrolyte or by enduring the large volume changes that some of the next-generation battery materials suffer greatly from.^26,27,149,150^ Considering the composition of the artificial SEI layer, it should be noted that the commercial LIBs are based on carbonate-based electrolytes, which react with the anode to form lithium alkyl carbonates.^151^ Hence, the lithium ethylene carbonate films grown by ALD/MLD using CO_2_ as the third precursor nicely mimic this composition.^127^ These films have not yet been characterized for their electrochemical performance, though.

So far, among ALD/MLD Li–organic thin films, ionic conductivity values have been investigated only for Li-ethylene glycol (3.6–5 × 10^–8^ S/cm at RT)^128^ and Li 1,4-benzenedisulfonate films (4.1 × 10^–9^ – 6.4 × 10^–8^ S/cm at 80–118 °C).^131^ The latter compound, Li-BDS, could be considered a prototype of so-called solid polymeric single Li-ion conductors with immobilized (sulfonate) anions; such materials have been highlighted as promising solid-state conductors for LIBs. In addition, a recent study on Li-GL films found them to be favorable for coating Li-metal anodes: The films were electrically insulating, ionically conductive, and relatively stable during cycling. A symmetrical Li/Li cell utilizing 60-layer Li-GL coatings survived over 13,000 Li-metal-electrode stripping/plating cycles without failure.^129^

Organic electrode materials are an attractive choice for use in batteries, as they are based of abundant and lightweight elements,^28,152^ and the all-solid-state thin-film microbattery form allows the two intrinsic drawbacks of these materials to be effectively circumvented, that is, their solubility in conventional liquid electrolytes and their poor conductivities. Moreover, in thin-film form, it is possible to investigate the intrinsic behaviors of the Li–organics, as no additives (such as conductive carbon) are needed.^28,132,133,153^ The ALD/MLD technique provides a uniquely suited approach to fabricate these materials as high-quality thin films,^23,25,28,52,132,154^ and even allows for the synthesis of alkali metal–organic materials not accessible by other techniques.^24,25,127,133^ The first all-ALD/MLD-made Li–organic microbattery consisted of a Li-benzoquinone cathode (importantly, in situ grown in its lithiated state) and Li-terephthalate anode, separated with an ALD-LiPON layer as the electrolyte.^52^ The battery worked also without the Li-terephthalate layer, the lithium metal layer intrinsically formed/consumed during the charge/discharge acting as an efficient anode. For these thin-film cells with ultrathin Li-benzoquinone and LiPON layers, ultrahigh redox reaction rates were realized;^52^ the charge/discharge times as short as ∼0.25 s (and energy/power densities of ∼100 mWh/cm^3^ and ∼500 W/cm^3^) are promising, considering that the setup was far from optimized yet.

Finally, moving to possible applications other than batteries, ferroelectric LiNbO_3_ films have been grown with ALD, and they exhibited a hysteresis loop and a coercive field of 220 kV/cm.^16^ Another example of the application potential is seen in the currently strongly highlighted field of MOF-type materials, considering that most of the alkali metal–organic films are readily grown in situ crystalline with ALD/MLD.^14,23,24,132,133^

## 8 Conclusions and Outlook

While alkali-metal-bearing processes do not yet form more than a niche among the research topics in the field of ALD technology, the interest in these processes is rapidly growing. This boost is driven by the role of the alkali metals in energy harvesting and storage technologies, the spearhead naturally being the lithium-ion battery technology. Accordingly, the Li-based processes are by far the most abundant among the alkali-metal ALD processes; in this decade-long era of alkali-metal ALD, close to 100 processes have been developed, out of which ca. 60 are for lithium as the metal component. Similarly, lithium has been the forerunner in the ALD/MLD research for hybrid alkali metal–organic thin films, started not much longer than five years ago.

The progress and development of Na, K, and Rb chemistries are scientifically very important and justified, but they still lack the revolutionary applications that would truly benefit from the thin-film form factor. We foresee that the full potential of manufacturing in situ crystalline thin-film alkali-metal MOFs is yet to be realized, as most of the studies have been focused on the ALD/MLD process development rather than strictly on their applications.

An especially promising approach is to apply Li-based ALD or ALD/MLD for the modification of electrode/solid–electrolyte interfaces in microbatteries. Inorganic materials provide a dense protective layer for the electrode, while the outer organic core provides a robust but flexible matrix that can withstand volume changes of the electrode. Another important approach is to utilize ALD and ALD/MLD thin-film materials as model electrodes for investigating their fundamental properties. Thin-film electrodes do not contain any additives, for example, binder or conductive carbon. Therefore, the electrodes consist only of the active material, and the performance metrics are intrinsic. This form factor is especially useful for studying phase changes during lithiation or in post-mortem analysis.

From the fundamental process development point-of-view, the monovalency and strong ionic character of the alkali metal species are both challenging and intriguing. Hence, processes with alkali metals are likely to reveal very interesting reaction pathways, which may still bear the essential criteria of an ALD or ALD/MLD process. The case of Li-HMDS is a prime example of this,^10^ where self-limiting physisorption or dissociative chemisorption become dominant based on the growth-terminating functional group. Here, we see a strong motivation for detailed mechanistic studies to shed in-depth light on these less straightforward ALD and ALD/MLD surface chemistries. Actually, similar features and film growth characteristics as seen for the alkali-metal-based processes could also play a (less pronounced) role in many “more conventional” ALD processes, though it is not fully recognized or acknowledged so far. Hence, deeper understanding and thereby utilization of these features could even open up new horizons for the rapidly expanding thin-film research field under the umbrella of ALD technology.

The mobility of the lightest alkali metals, in particular lithium, poses other issues as well in the process control. On the other hand, it also provides us with new possibilities, namely, lithium has been successfully ex situ introduced into ternary or quaternary materials with a high degree of control. This is somewhat similar to the so-called vapor phase infiltration approach utilized for the incorporation of inorganic ALD precursors into porous polymer-type matrices.^155^

Practical difficulties in the research on alkali-metal-bearing processes often arise from the fact that many of the alkali metal compounds characteristically tend to react with H_2_O and CO_2_. Another issue is the so-called reservoir effect, where typically LiOH forms stable hydrates during the H_2_O pulse. Water, upon desorption, results in nonsurface limited growth, which is often seen as irregular growth. This complex behavior makes the process optimization sometimes difficult. Here, the choice of the optimal combination of precursors could help to diminish the problem.

Finally, particularly in ALD/MLD, an exciting benefit has been seen in the fact that the ionic character of the bonds in alkali metal compounds apparently promotes the in situ crystallization of the resultant thin films. This is interesting from the scientific point of view, as it has already enabled the fabrication of several previously unknown metal–organic network materials, and a lot more could be expected in this direction. Also, crystalline films are important for various applications. Combining alkali metals with organics, especially organic acids, typically results in rather very stable materials that do not absorb moisture. Here, a yet-unexplored playground could be to combine organics (even several different ones) with the alkali metal component, not only in the ordinary 1:1 ratio, but any arbitrary ratio for various superlattices or gradient materials where the organics could bring to the original alkali metal compound functionalities such as mechanical flexibility, optical controllability, etc.^134,156−158^
